# Development and validation of a prediction model for unsuccessful treatment outcomes in patients with multi-drug resistance tuberculosis

**DOI:** 10.1186/s12879-023-08193-0

**Published:** 2023-05-05

**Authors:** J-B Ma, L-C Zeng, F Ren, L-Y Dang, H Luo, Y-Q Wu, X-J Yang, R Li, H Yang, Y Xu

**Affiliations:** 1 Department of Drug-resistance tuberculosis, Xi’an Chest Hospital, Xi’an, Shaanxi Province China; 2 Xi’an Center for Disease Control and Prevention, Xi’an, Shaanxi Province China; 3 Department of Clinical Laboratory, Xi’an Chest Hospital, Xi’an, Shaanxi Province China

**Keywords:** Tuberculosis, Drug resistance, Treatment outcome, Nomogram, Prediction

## Abstract

**Background:**

The World Health Organization has reported that the treatment success rate of multi-drug resistance tuberculosis is approximately 57% globally. Although new drugs such as bedaquiline and linezolid is likely improve the treatment outcome, there are other factors associated with unsuccessful treatment outcome. The factors associated with unsuccessful treatment outcomes have been widely examined, but only a few studies have developed prediction models. We aimed to develop and validate a simple clinical prediction model for unsuccessful treatment outcomes in patients with multi-drug resistance pulmonary tuberculosis (MDR-PTB).

**Methods:**

This retrospective cohort study was performed between January 2017 and December 2019 at a special hospital in Xi’an, China. A total of 446 patients with MDR-PTB were included. Least absolute shrinkage and selection operator (LASSO) regression and multivariate logistic regression were used to select prognostic factors for unsuccessful treatment outcomes. A nomogram was built based on four prognostic factors. Internal validation and leave-one-out cross-validation was used to assess the model.

**Results:**

Of the 446 patients with MDR-PTB, 32.9% (147/446) cases had unsuccessful treatment outcomes, and 67.1% had successful outcomes. After LASSO regression and multivariate logistic analyses, no health education, advanced age, being male, and larger extent lung involvement were identified as prognostic factors. These four prognostic factors were used to build the prediction nomograms. The area under the curve of the model was 0.757 (95%CI 0.711 to 0.804), and the concordance index (C-index) was 0.75. For the bootstrap sampling validation, the corrected C-index was 0.747. In the leave-one-out cross-validation, the C-index was 0.765. The slope of the calibration curve was 0.968, which was approximately 1.0. This indicated that the model was accurate in predicting unsuccessful treatment outcomes.

**Conclusions:**

We built a predictive model and established a nomogram for unsuccessful treatment outcomes of multi-drug resistance pulmonary tuberculosis based on baseline characteristics. This predictive model showed good performance and could be used as a tool by clinicians to predict who among their patients will have an unsuccessful treatment outcome.

**Supplementary Information:**

The online version contains supplementary material available at 10.1186/s12879-023-08193-0.

## Background

Tuberculosis (TB) is an infectious disease caused by *Mycobacterium tuberculosis*. Approximately 10 million newly diagnosed cases of tuberculosis and 1.5 million deaths are reported every year by the World Health Organization [1]. China has one of the highest burdens of tuberculosis, with about 0.9 million people diagnosed with tuberculosis every year [1]. Therefore, measures must be implemented to prevent people from getting TB. However, multi-drug resistance tuberculosis (MDR-TB) remains a challenge.

MDR-TB is defined as *M.tuberculosis* resistance to isoniazid and rifampicin. The treatment of MDR-TB often requires more than four second-line anti-TB drugs over 18–24 months [2]. The treatment of MDR-TB burdens families with high healthcare expenses [3, 4], and patients can suffer from severe side effects [5, 6]. The current treatments lead to only 54% of patients with MDR-TB being successfully treated, with lower success rates in low-income countries [1]. The success rate is lower than 54% in China [1, 7]. Prediction models can help clinicians identify patients at risk of unsuccessful treatment outcomes. Clinicians can take measures like strengthening health education, call for more social and family support to increase treatment success.

Many risk factors associated with unsuccessful treatment outcomes of MDR-TB have been reported, including age, sex, smoking, alcohol abuse, diabetes, HIV infection, and treatment history [5, 8–11]. A prediction model for the treatment outcomes of sensitive tuberculosis has been developed in China [12–14]. However, few studies have developed models to predict the treatment outcomes of MDR-TB. We conducted a cohort study at Xi’an Chest Hospital from 2017 to 2019. Our goal was to develop a prediction model to help clinicians identify patients with MDR pulmonary tuberculosis (MDR-PTB) at risk of unsuccessful treatment outcomes.

## Methods

### Patients and study design

This was a retrospective cohort study involving patients diagnosed with MDR-PTB from Jan 2017 to Dec 2019 at Xi’an Chest Hospital. Patients were treated according to the *Treatment Guidelines for Drug Resistant Tuberculosis, 2016 update* for 18 to 24 months [9]. The characteristics of patients were shown in Table S1. From2017 to 2019 new drugs like Bedaquiline and Delamanid was not used in our hospital, and government funding was not changed, also all second line drugs (including Fluoroquinolones, Linezolid, Bedaquiline, Clofazimine, Cycloserine, Injectable agents) were not free for patients. Patients who died before the start of MDR-TB treatment, received an incorrect regimen(lack enough core medication), were co-infected with non-tuberculosis mycobacterium, or received a short-term regimen were excluded.

Data were collected from the electronic medical record system, including population characteristics (sex, age, height, weight, marriage, address), co-morbidities (diabetes and HIV), laboratory test results (sputum smear test, drug sensitive test), chest radiographic findings (cavity and lung lesion distribution), and treatment regimens. Data were managed using the EpiData system and entered by individuals. To minimize bias, the data and outcomes that had been entered were checked by attending doctors.

Chest radiographs taken at the beginning of MDR-TB treatment were used to assess the lung lesion distribution. All radiographs were reviewed by a radiologist. Post-anterior chest radiographs were divided into six fields by two horizontal lines at the lower edge of the second and fourth ribs. The lung lesion distribution was assessed based on the number of lung lesion fields. One lung lesion field was counted as long as there is a lesion in one lung field, instead of adding up the area of the lesion.

Patients diagnosed in 2019 received systematic health education under the guidance of Family Health International (FHI 360). All patients were hospitalized during the beginning of treatment, all patients received health education. Patients were educated on MDR-TB, the importance of regular treatment, probable drug side effects and management methods, drug dosage and frequency, and treatment duration. We presume patients enrolled before 2019 was not given health education because it was not systematic and specific previously.

### Definitions

Tuberculosis was classified into new case tuberculosis and previously treated tuberculosis according to treatment history. New case for tuberculosis was defined as tuberculosis that had never been treated with anti-tuberculosis drugs or was treated for less than one month. Previously treated tuberculosis includes tuberculosis that has been treated for over one month, relapsed, or failed to respond to the initial treatment administered.

Treatment outcomes were classified into six categories [15]. The first outcome was defined as cured when patients completed the treatment regimen and had three consecutive negative cultures taken at least 30 days apart after the intensive phase. Second, ‘treatment completion’ means patients who completed the treatment regimen, had three consecutive negative cultures and no evidence of treatment failure. The third outcome was defined as treatment failure when the treatment was terminated, the treatment regimen was changed to at least two drugs due to drug side effects, the culture remained positive by the end of the intensive phase, the culture reverted in the continuation phase, or the patient acquired resistance to fluoroquinolones or injectable agents (kanamycin, amikacin, or capreomycin). The fourth outcome was defined as a loss to follow-up when the treatment was interrupted for more than two months. Fifth, death was defined as death from any reason during the treatment. The sixth outcome was that the patient was transferred to another hospital during treatment. Outcomes were reclassified as treatment success (including cure and treatment completion) and unsuccessful treatment outcomes (including treatment failure, lost to follow-up, and death) [15].

### Laboratory cultures and antibiotics sensitivity test

The BACTEC MGIT 960 culture system (Becton, Dickinson and Company, America) was used for mycobacterial culture. For positive results, strain identification was performed using an Mpb64 monoclonal antibody(Hangzhou Genesis Biodetecton & Biocontrol Ltd, China). First-line drug sensitivity was tested using the BACTEC MGIT 960 system, and second-line drug sensitivity was tested using the absolute concentration method. The drug concentrations for isoniazid, rifampin, levofloxacin, moxifloxacin, amikacin, and capreomycin were 0.1, 1.0, 2.0, 1.0, 30, 40 µg/mL, respectively. The Ziehl-Neelsen acid-fast staining was used for the smear test.

### Data analysis

Data analysis was performed using R version 3.3.2 (http://www.R-project.org, The R Foundation) and Free Statistics version 1.5. A P-value of < 0.05 (two-sided) was considered to be statistically significant. The patients characteristics are shown according to treatment outcomes (success and unsuccessful treatment outcomes) in table one. Normally distributed continuous variables are presented as means with standard deviations (SD) and non-normally distributed continuous variables as medians with an interquartile range (IQR). Categorical variables were expressed as the number and percentage of patients in each category. Continuous variables were compared using the *t-*test for variables with a normal distribution and the Mann-Whitney *U* test for variables with an abnormal distribution. Proportions were compared using the χ2 test with Yates correction or Fisher exact test. Twenty-four cases missing data for fluoroquinolone and injectable agent drug sensitive test were inserted according to imputed. According to the resistance rate, there are maybe eight cases and one case fluoroquinolone resistance and injectable agent drug resistance. Cases which were older and have longer usage of these drugs were inserted with resistant.

Several steps were performed to develop and validate nomograms for predicting unsuccessful treatment outcomes. First, least absolute shrinkage and selection operator (LASSO) regression was conducted to select the potential prognostic factors. Second, multivariable logistic regression analysis was performed to identify the significant prognostic factors associated with unsuccessful treatment outcomes. Third, all potential prognostic factors were used to build the model, and a nomogram was used to visualize the model. Receiver operating characteristic (ROC), concordance index (c-index), and calibration curve analyses were used to evaluate the discrimination and calibration of the model. Furthermore, the corrected ROC, C-index, and calibration curve analyses were calculated using 1000 bootstrap sampling. To evaluate the performance of the model further, leave-one-out cross-validation was performed.

## Results

**Fig. 1:**
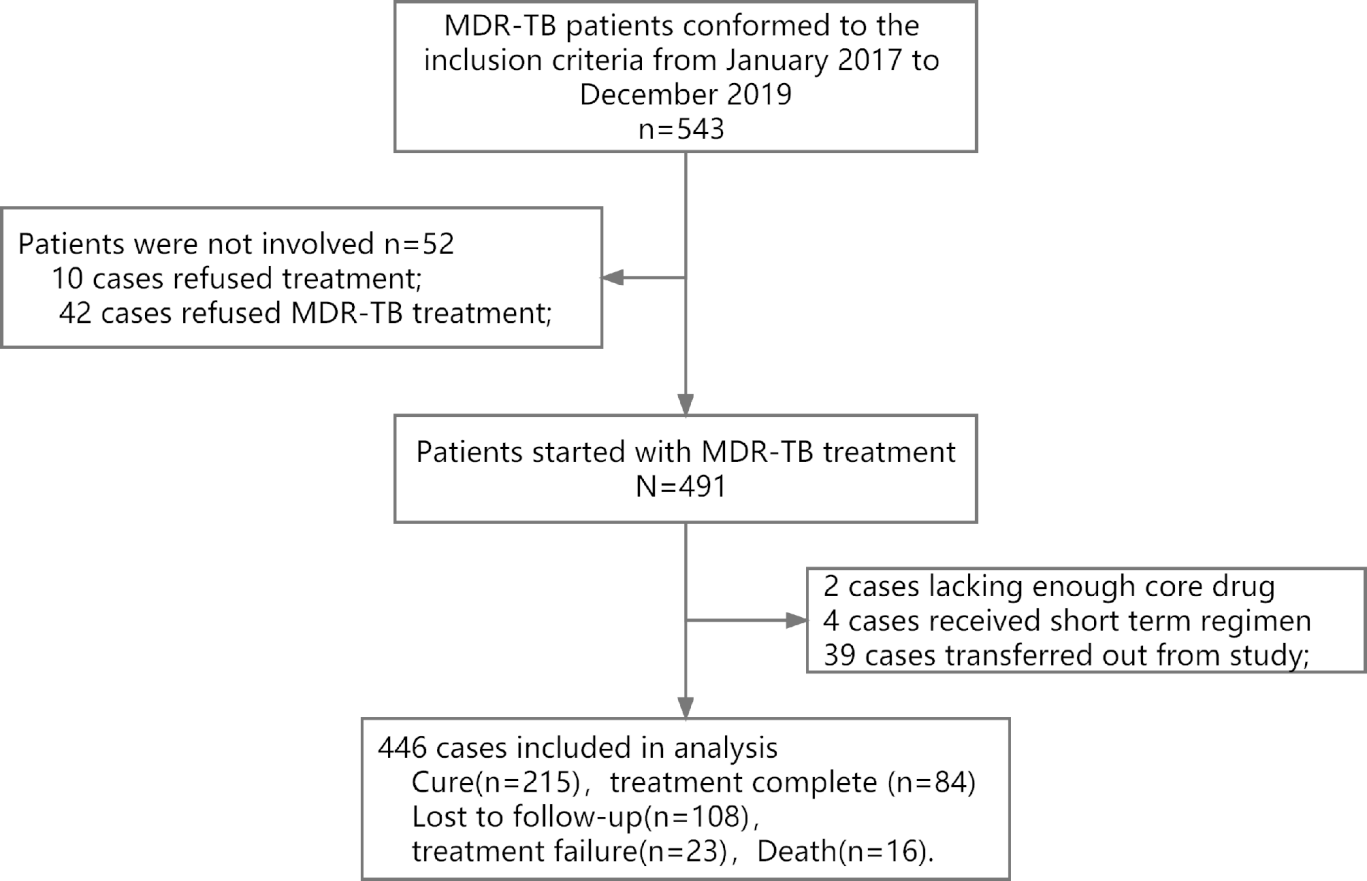
Flow chart of the study participants. The box shows the details of each node, and the The connection line shows the exclude process. Abbreviations: MDR-TB: multi-drug resistance tuberculosis

A total of 543 patients were diagnosed with MDR-PTB at Xi’an Chest Hospital between January 2017 and December 2019 (Fig. 1). Additionally, 97 patients were excluded (ten refused treatment, 42 refused MDR-TB treatment, two lacked core drugs, four received a short-term regimen, and 39 were transferred out of the study). Finally, 446 cases were included in the analysis, with 299 (67%) cases of successful treatment outcomes and 147 (33%) cases of unsuccessful treatment outcomes.

Table 1 shows the characteristics of the successful and unsuccessful treatment outcome groups. The median age of the included patients was 38.1 ± 15.0 years, and 70% (312/446) were male. In total, 264 (59.2%) patients had positive sputum/BALF smears, 110 (24.7%) were infected with *M.tuberculosis* resistant to fluoroquinolones, and two were HIV-positive. Health education, sex, age, marriage status, sputum/BALF smear, lung lesion distribution, treatment history, and history of diabetes mellitus were significantly different between the two groups.

**Table 1:** Clinical characteristics and drugs used in the successful and unsuccessful treatment outcome groups

Variables	Total (n = 446)	Success (n = 299)	UTO (n = 147)	*P*
Health education ^a^	225 (50.4)	168 (56.2)	57 (38.8)	< 0.001
Sex ^a^				< 0.001
female	134 (30.0)	109 (36.5)	25 (17)	
male	312 (70.0)	190 (63.5)	122 (83)	
Age(years)^b^	38.1 ± 15.0	34.9 ± 13.8	44.7 ± 15.4	< 0.001
Address ^a^				0.653
Xi’an	189 (42.4)	124 (41.5)	65 (44.2)	
Other regions	257 (57.6)	175 (58.5)	82 (55.8)	
BMI(kg/m^2^)^b^	20.3 ± 2.8	20.3 ± 2.8	20.1 ± 2.9	0.509
Marriage ^a^				0.03
married	292 (65.5)	185 (61.9)	107 (72.8)	
single	154 (34.5)	114 (38.1)	40 (27.2)	
Grade of smear ^a^				0.007
0	182 (40.8)	137 (45.8)	45 (30.6)	
1	106 (23.8)	72 (24.1)	34 (23.1)	
2	53 (11.9)	31 (10.4)	22 (15)	
3	49 (11.0)	30 (10)	19 (12.9)	
4	56 (12.6)	29 (9.7)	27 (18.4)	
**Drug sensitivity test**				
Fluoroquinolones ^a^				0.448
sensitive	336 (75.3)	229 (76.6)	107 (72.8)	
resistant	110 (24.7)	70 (23.4)	40 (27.2)	
Injectable agents ^a^				0.902
sensitive	430 (96.4)	289 (96.7)	141 (95.9)	
resistant	16 ( 3.6)	10 (3.3)	6 (4.1)	
Cavity ^a^	285 (63.9)	195 (65.2)	90 (61.2)	0.471
Lung Lesions (field) ^b^	3.1 ± 1.8	2.8 ± 1.7	3.8 ± 1.8	< 0.001
Treatment history ^a^				< 0.001
New case	236 (52.9)	176 (58.9)	60 (40.8)	
Previously treated	210 (47.1)	123 (41.1)	87 (59.2)	
Diabetes mellitus ^a^	70 (15.7)	35 (11.7)	35 (23.8)	0.002
**Drugs used in regimen**				
Fluoroquinolones ^a^	373 (83.6)	255 (85.3)	118 (80.3)	0.227
Linezolid ^a^	113 (25.3)	74 (24.7)	39 (26.5)	0.771
Cycloserine ^a^	332 (74.4)	228 (76.3)	104 (70.7)	0.255
Clofazimine ^a^	36 ( 8.1)	22 (7.4)	14 (9.5)	0.546
Ethambutol ^a^	181 (40.6)	124 (41.5)	57 (38.8)	0.658
Pyrazinamid ^a^	439 (98.4)	294 (98.3)	145 (98.6)	1
injectable agents ^a^	431 (96.6)	290 (97)	141 (95.9)	0.582
Prothionamide ^a^	406 (91.0)	271 (90.6)	135 (91.8)	0.809
P-aminosalicylate ^a^	30 ( 6.7)	16 (5.4)	14 (9.5)	0.146

a, n (%),b, Mean ± SD. UTO, unsuccessful treatment outcome

**Fig. 2:**
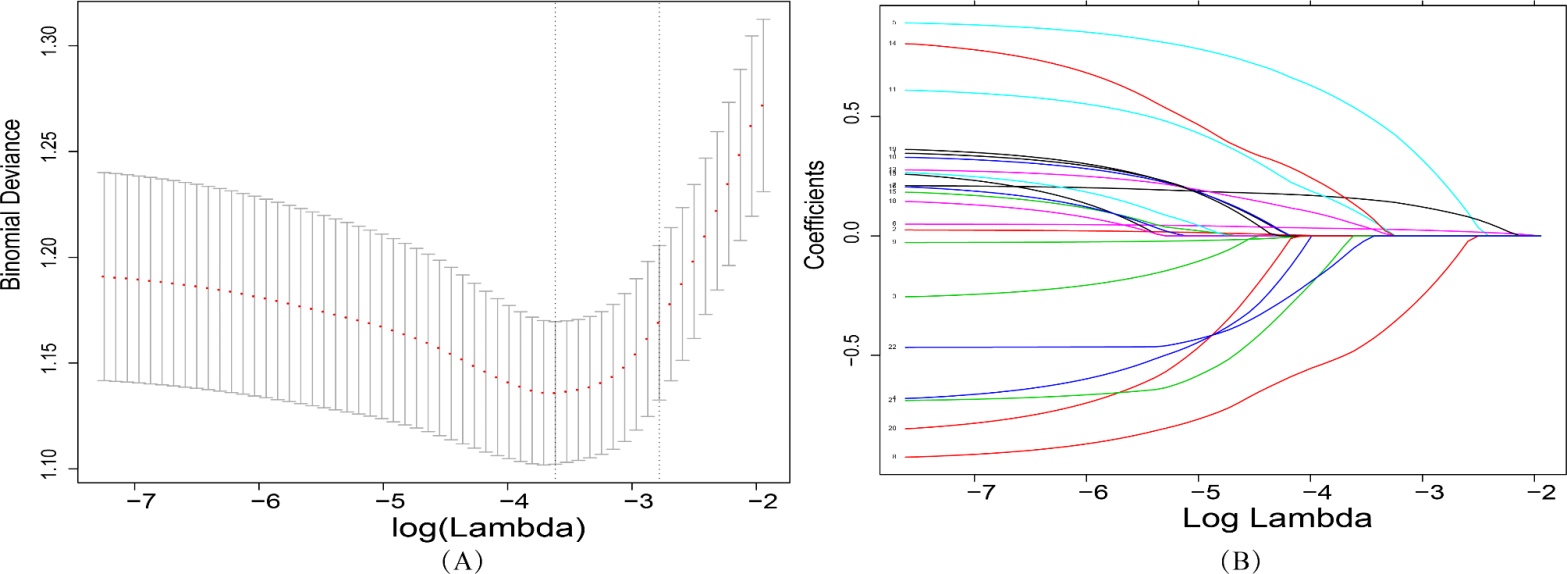
LASSO regression plot. (A) Plot of partial likelihood deviance; (B) plot of LASSO coefficient profiles. Each curve represents the LASSO coefficient profile of a feature against the log (lambda) sequence. when the optimal lambda value was 0.062, retentiong variables were screen

LASSO regression analysis was performed to identify the potential prognostic factors. Figure 2 shows that when the optimal lambda value was 0.062, no health education, old age, male, and larger extent of lung lesion distribution were associated with unsuccessful treatment outcomes. These factors were included in a logistic regression analysis (Table 2), which showed that the odds ratio (OR) for unsuccessful treatment outcomes was 2.43 (1.44–4.1) for males compared to females. The OR for unsuccessful treatment outcomes was 2.27 for no health education compared with health education. It also shows that when one’s age increases by one year, the incidence of unsuccessful treatment outcomes increases by 4%. In addition, the incidence was found to increase by 25% when the lung lesion distribution increases by one field.

**Table 2:** Multivariable logistic analyses of the risk factors and UTO

Variables	Crude model		Adjusted model	
	OR(95%*Cl*)	*P* value	Adjusted odds ratios	*P* value
Sex				
female	reference		reference	
male	2.8 (1.71–4.57)	< 0.001	2.43 (1.44–4.1)	0.001
Health education				
NO	reference		reference	
Yes	0.49 (0.33–0.74)	0.001	0.44 (0.28–0.69)	< 0.001
Lung Lesions (field)	1.37 (1.23–1.54)	< 0.001	1.25 (1.1–1.41)	0.001
Age	1.05 (1.03–1.06)	< 0.001	1.04 (1.03–1.06)	< 0.001

Abbreviations: UTO, unsuccessful treatment outcome;OR,odds ratios

**Fig. 3:**
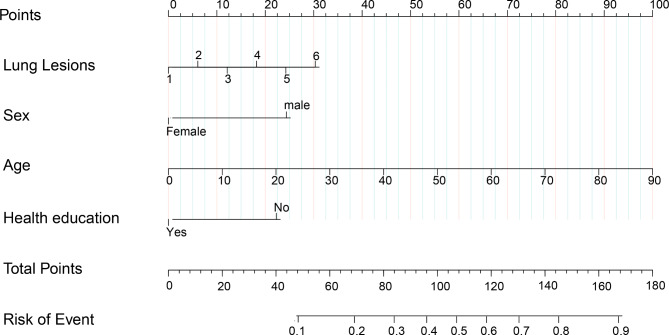
Nomogram to predict the probability of unsuccessful treatment outcomes in patients with MDR-PTB. According to nomogram points for lung lesions, sex, age, Health education can be calculated from first line. Total points were the sum of the four points. And we can evaluate the risk of unsuccessful treatment outcomes from the seventh line according to the total point

**Fig. 4:**
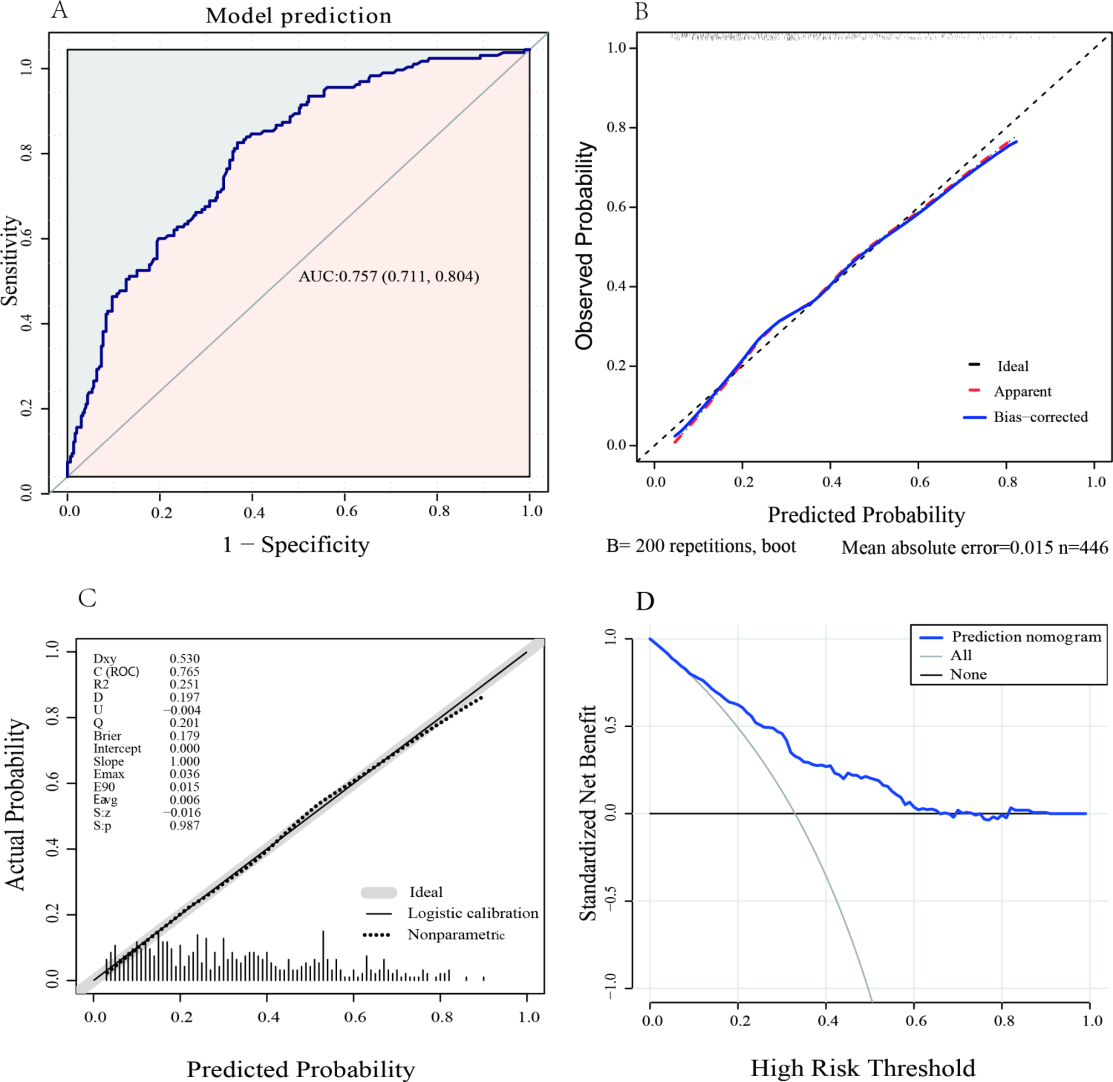
The discrimination and calibration assessment of the model **(A)** ROC curve and AUC of the nomogram in the training cohort. **(B)** Calibration curve for the nomogram to predict the probability of unsuccessful treatment outcomes with bootstrap sampling validation. **(C)** Calibration curve for the nomogram to predict the probability of unsuccessful treatment outcome with leave-one-out cross-validation. **(D)** Decision curve for the predictive nomogram. The net benefits were measured at different threshold probabilities. The blue line represents the predictive nomogram. The gray line represents the assumption that all patients have unsuccessful treatment outcomes. The black line represents the assumption that no patients have unsuccessful treatment outcomes

Based on multivariate logistic regression analyses, four prognostic factors were used to establish the nomogram (Fig. 3). The nomogram was assessed using the area under the curve (AUC), concordance index (C-index), and calibration curve. As shown in Fig. 4A, the AUC for predicting unsuccessful treatment outcomes was 0.757 (95%CI 0.711 to 0.804); when the Yoden index was 0.382, it had a sensitivity of 0.640 and specificity of 0.773. The concordance index (C-index) of the model was 0.75. Bootstrap sampling validation was performed, and the corrected C-index was 0.747. As shown in Fig. 4B, to assess the accuracy of the model, a calibration curve was drawn, and the slope of the calibrate was 0.968, close to 1.0. In Fig. 4C, we performed leave-one-out cross-validation to evaluate the model. In this, the corrected C-index was 0.765, and the slope of the calibration was 1.0. Figure 4D shows the decision curve analysis. This analysis showed that patients could benefit from the model when the threshold probabilities were 0.086–0.713.

## Discussion

In this study, We developed a simple prognostic nomogram using factors include age, sex, health education, and lung lesion distribution. This nomogram was assessed using the C-index, AUC, and calibration curve and showed good performance and accuracy in predicting unsuccessful treatment outcomes. This nomogram can help clinicians predict the probability of unsuccessful treatment outcomes in patients with MDR-PTB. For example, a 60 year old man with a 4 lung lesion distribution and have no health education, in the nomogrames 60 year score for age is 65 point, being male score 25 point, 4 lung lesion distribution score 18 point, no health education score 24 point, the total point would be 132 point, which would indicate this old man may have a 70% risk of unsuccessful treatment outcome.

Researchers have developed prediction models to help clinicians identify patients at a high risk of unsuccessful treatment outcomes. A study based on 802 patients in Brazil developed a model using four factors (including the number of previous MDR-TB treatments, how the case was discovered, smoking and treatment type), with an AUC of 0.76 [16]. Another study conducted in Hunan, China, illustrated that a history of second-line TB treatment, resistance to any fluoroquinolones, and smear that did not convert from positive to negative by two months was a prognostic factor for unsuccessful ltreatment outcomes and developed a prediction model with a moderate AUC [17]. However, these studies had moderate performance or were not well validated. In addition, it is difficult to accurately determine factors such as treatment history, smoking, and smear conversion time.

Of the patients with MDR-PTB involved in our study, 52.9% were identified with new case tuberculosis, a little higher than 48.1% reported in a national survey in China [18] and lower than that reported in Japan (62%) [10]. The resistance rate to fluoroquinolone was 24.7%, similar to the 2020 Global Tuberculosis Report, which reported a fluoroquinolone resistance rate of 20.1% [19], and to the rates reported in other studies in China (17.3–23.4%) [20, 21]. Furthermore, 14% of patients had diabetes mellitus, a little higher than that reported in other studies (8–12%) [8, 22 to 20]. The treatment success rate was 67%, the same as reported in other regions of China (57-75%) [11, 23, 24] and other countries [5, 25, 26]. These characteristics indicate that our prognostic model may apply to patients in other regions.

The prevalence of tuberculosis and MDR-TB is higher in males than in females. In our study, 70% of the patients were male, which corroborated the results of previous studies [5, 23, 24, 27]. Previous studies have shown that the male sex is a risk factor for unsuccessful outcomes [28]. In our study, males had 2.43 times higher odds of unsuccessful treatment outcomes than females. Male patients may have poor adherence to drug use and follow-up. Male patients also tend to have a higher smoking rate, which has been reported as a risk factor for unsuccessful treatment outcomes [5]. We found that a year increase in age led to a 4% increase in unsuccessful treatment outcomes. Previous studies have reported that unsuccessful treatment outcomes are associated with age [10, 11, 25, 28]. This may be because older people have more comorbidities, lack economic support, and suffer more drug side effects [9].

Health education is important in the management of long-term diseases. We conducted a systematic health education program under the guidance of the Family Health International (FHI 360). Previous studies have found that low education is a risk factor for treatment interruption of tuberculosis [29, 30]. A study from the Philippines reported that better general TB knowledge could protect patients with MDR-TB from being lost to follow-up [31]. A meta-analysis showed that patient education was associated with a lower rate of treatment default [32]. Some patients with MDR-TB have low education and income levels and lack knowledge about tuberculosis. Therefore, health education should be provided to patients with MDR-TB. As shown in our study, no health education patients had 1.56 times higher odds of unsuccessful treatment outcomes than patients who received heath education. Therefore, we hope that systematic health education will become more widely conducted. Studies have found unsuccessful outcomes associated with cavities in the chest and high-grade smears [23, 25]. We found that smear grade and lung lesion distribution may be associated with unsuccessful treatment outcomes in a univariate analysis. This indicates that serious tuberculosis may have a high rate of unsuccessful treatment outcomes.

Our model has a few advantages. First, the four factors used in the model are baseline characteristics that can be acquired easily. Second, our model showed good discrimination and was found to be accurate using the AUC and calibration. Third, our model was validated by bootstrap sampling and leave-one-out cross-validation, with both showing good performance.

This study has several limitations. First, because of this was an observational study confounding cannot be completely excluded and other time-varying factors might have been confounding and this does not prove causality. Second, this study was conducted in one hospital, and the model was not externally validated, which may have a regional limitation. Third, New drugs include Bedaquiline and Delamanid were not uses in this study, this prediction model does not apply to the newer regimens. As newer drugs are not always available, especially in the west of China, this prediction model may be of use to clinicians where patients are treated with the included drugs in this study. In addition, HIV co-infection was not analyzed because there were only two cases of HIV positivity, and this model cannot be used in patients that are HIV-positive. Mpb64 monoclonal antibody was not a recommended method of Mtb identification. systemic health education has not yet been widely conducted. Despite these limitations, we developed an accurate predictive model for unsuccessful treatment outcomes in patients with MDR-TB. We hope that this predictive nomogram can help clinicians improve the outcomes of patients with MDR-TB.

## Conclusions

A predictive model and nomogram for predicting unsuccessful treatment outcomes in patients with MDR-PTB were built and showed good discrimination and calibration. Our model can help clinicians better manage MDR-PTB cases.

## Electronic supplementary material

Below is the link to the electronic supplementary material.

Supplementary Material 1 Table S1. The characteristics of patients from 2017 to 2019.

## Data Availability

The datasets used and/or analyzed during the current study are not publicly available due restrictions of hospital rule, but are available from the corresponding author on reasonable request.
