# Supplementary material for: Development and validation of a prediction model for unsuccessful treatment outcomes in patients with multi-drug resistance tuberculosis
Source: BMC Infect Dis. 2023 May 5;23:289. doi: 10.1186/s12879-023-08193-0 (PMC10161636; doi:10.1186/s12879-023-08193-0)
Supplement: Supplementary file 1 — Supplementary Material 1 Table S1. The characteristics of patients from 2017 to 2019. [file 12879_2023_8193_MOESM1_ESM.docx]

Table S1. The characteristics of patients from 2017 to 2019.

| Variables | Total  (n = 446) | 2017  (n = 101) | 2018  (n = 120) | 2019  (n = 225) | p |
| --- | --- | --- | --- | --- | --- |
| health education | 225 | 0 | 0 | 225 |  |
| sex, n (%) |  |  |  |  | 0.414 |
| female | 134 (30.0) | 27 (26.7) | 33 (27.5) | 74 (32.9) |  |
| male | 312 (70.0) | 74 (73.3) | 87 (72.5) | 151 (67.1) |  |
| age, Mean ± SD | 38.1 ± 15.0 | 38.0 ± 14.8 | 36.4 ± 14.9 | 39.1 ± 15.1 | 0.286 |
| Address, n (%) |  |  |  |  | 0.538 |
| Xi'an | 189 (42.4) | 41 (40.6) | 56 (46.7) | 92 (40.9) |  |
| Other regions | 257 (57.6) | 60 (59.4) | 64 (53.3) | 133 (59.1) |  |
| BMI, Mean ± SD | 20.3 ± 2.8 | 20.7 ± 2.6 | 20.3 ± 3.1 | 20.1 ± 2.8 | 0.26 |
| Marriage, n (%) |  |  |  |  | 0.136 |
| married | 292 (65.5) | 73 (72.3) | 81 (67.5) | 138 (61.3) |  |
| single | 154 (34.5) | 28 (27.7) | 39 (32.5) | 87 (38.7) |  |
| Grade.of.smear, n (%) |  |  |  |  | < 0.001 |
| 0 | 182 (40.8) | 28 (27.7) | 52 (43.3) | 102 (45.3) |  |
| 1 | 106 (23.8) | 46 (45.5) | 16 (13.3) | 44 (19.6) |  |
| 2 | 53 (11.9) | 14 (13.9) | 19 (15.8) | 20 (8.9) |  |
| 3 | 49 (11.0) | 4 (4) | 17 (14.2) | 28 (12.4) |  |
| 4 | 56 (12.6) | 9 (8.9) | 16 (13.3) | 31 (13.8) |  |
| **Drug sensitivity test** |  |  |  |  |  |
| Fluoroquinolones, n (%) |  |  |  |  | < 0.001 |
| sensitive | 336 (75.3) | 58 (57.4) | 82 (68.3) | 196 (87.1) |  |
| resistant | 110 (24.7) | 43 (42.6) | 38 (31.7) | 29 (12.9) |  |
| Injectable.agents, n (%) |  |  |  |  | 0.01 |
| sensitive | 430 (96.4) | 93 (92.1) | 115 (95.8) | 222 (98.7) |  |
| resistant | 16 ( 3.6) | 8 (7.9) | 5 (4.2) | 3 (1.3) |  |
| Cavity, n (%) | 285 (63.9) | 57 (56.4) | 75 (62.5) | 153 (68) | 0.124 |
| Lung.Lesions..field., n (%) |  |  |  |  | 0.011 |
| 1 | 106 (23.8) | 11 (10.9) | 28 (23.3) | 67 (29.8) |  |
| 2 | 101 (22.6) | 24 (23.8) | 34 (28.3) | 43 (19.1) |  |
| 3 | 68 (15.2) | 14 (13.9) | 17 (14.2) | 37 (16.4) |  |
| 4 | 45 (10.1) | 13 (12.9) | 12 (10) | 20 (8.9) |  |
| 5 | 53 (11.9) | 15 (14.9) | 9 (7.5) | 29 (12.9) |  |
| 6 | 73 (16.4) | 24 (23.8) | 20 (16.7) | 29 (12.9) |  |
| Treatment.history, n (%) |  |  |  |  | < 0.001 |
| New case | 236 (52.9) | 35 (34.7) | 70 (58.3) | 131 (58.2) |  |
| Previously treated | 210 (47.1) | 66 (65.3) | 50 (41.7) | 94 (41.8) |  |
| Diabetes.mellitus, n (%) | 70 (15.7) | 12 (11.9) | 16 (13.3) | 42 (18.7) | 0.21 |
| **Drugs used in regimen** |  |  |  |  |  |
| Fluoroquinolones, n (%) | 373 (83.6) | 84 (83.2) | 101 (84.2) | 188 (83.6) | 0.979 |
| Cycloserine, n (%) | 332 (74.4) | 67 (66.3) | 90 (75) | 175 (77.8) | 0.09 |
| Clofazimine, n (%) | 36 ( 8.1) | 8 (7.9) | 4 (3.3) | 24 (10.7) | 0.059 |
| Linezolid, n (%) | 113 (25.3) | 17 (16.8) | 20 (16.7) | 76 (33.8) | < 0.001 |
| Ethambutol, n (%) | 181 (40.6) | 46 (45.5) | 53 (44.2) | 82 (36.4) | 0.195 |
| Pyrazinamid, n (%) | 439 (98.4) | 99 (98) | 117 (97.5) | 223 (99.1) | 0.476 |
| Prothionamide, n (%) | 406 (91.0) | 95 (94.1) | 115 (95.8) | 196 (87.1) | 0.013 |
| P.aminosalicylate, n (%) | 30 ( 6.7) | 12 (11.9) | 10 (8.3) | 8 (3.6) | 0.015 |
